# Serology- and PCR-based cumulative incidence of SARS-CoV-2 infection in adults in a successfully contained early hotspot (CoMoLo study), Germany, May to June 2020

**DOI:** 10.2807/1560-7917.ES.2020.25.47.2001752

**Published:** 2020-11-26

**Authors:** Claudia Santos-Hövener, Hannelore K Neuhauser, Angelika Schaffrath Rosario, Markus Busch, Martin Schlaud, Robert Hoffmann, Antje Gößwald, Carmen Koschollek, Jens Hoebel, Jennifer Allen, Antje Haack-Erdmann, Stefan Brockmann, Thomas Ziese, Andreas Nitsche, Janine Michel, Sebastian Haller, Hendrik Wilking, Osamah Hamouda, Victor M Corman, Christian Drosten, Lars Schaade, Lothar H Wieler, CoMoLo Study Group, Thomas Lampert, CoMoLo Study Group, CoMoLo Study Group, Stefan Albrecht, Sabine Born, Hans Butschalowsky, Nina Buttmann-Schweiger, Stefan Damerow, Ute Ellert, Julia Fiebig, Andrea Franke, Julian Gräf, Jasmin Gundlach, Isabell Hey, Sebastian Hinck, Marcel Hintze, Heike Hölling, Robin Houben, Antje Hüther, Melanie Krugmann, Ulrike Kubisch, Ronny Kuhnert, Tim A. Kuttig, Michael Lange, Stefan Meisegeier, Stephan Müters, Ruth Offergeld, Hanna Perlitz, Christina Poethko-Müller, Ute Pöplow do Rego, Franziska Prütz, Anna Sandoni, Giselle Sarganas, Gina Schöne, Silke Stahlberg, Julia Strandmark, Roma Thamm, Felicitas Vogelgesang, Benjamin Wachtler, Jörg Wernitz, Matthias Wetzstein, Christin Wolff

**Affiliations:** 1 Robert Koch Institute, Berlin, Germany; 2 These authors contributed equally; 3 Landratsamt Hohenlohekreis, Gesundheitsamt, Künzelsau, Germany; 4 Department of Health Protection and Epidemiology, Baden-Wuerttemberg State Health Office, Stuttgart, Germany; 5 National Consultant Laboratory for Coronaviruses, Berlin Institute of Virology, Charité - Universitätsmedizin, Berlin, Germany; 6 German Centre for Infection Research (DZIF), Berlin, Germany; 7 The members of the group are listed under Investigators

**Keywords:** SARS-CoV-2, COVID-19, seroepidemiologic studies, seroprevalence, antibody

## Abstract

Three months after a coronavirus disease (COVID-19) outbreak in Kupferzell, Germany, a population-based study (n = 2,203) found no RT-PCR-positives. IgG-ELISA seropositivity with positive virus neutralisation tests was 7.7% (95% confidence interval (CI): 6.5–9.1) and 4.3% with negative neutralisation tests. We estimate 12.0% (95% CI: 10.4–14.0%) infected adults (24.5% asymptomatic), six times more than notified. Full hotspot containment confirms the effectiveness of prompt protection measures. However, 88% naïve adults are still at high COVID-19 risk.

After a large church concert on 1 March 2020 and a first detected infection with severe acute respiratory syndrome coronavirus 2 (SARS-CoV-2) on 9 March, the southern German community of Kupferzell in the federal state Baden-Württemberg faced a steep increase of SARS-CoV-2 infections. Investigations of the local health authorities showed increasing evidence of community spreading in a complex and chronologically dense pattern of travel returnees who attended a choir and trombone church concert. Wide-reaching infection prevention and local control measures were implemented starting in the week of the first case detection, followed by additional measures such as a ban on gatherings in the federal state starting mid-March. The number of SARS-CoV-2 infections peaked in March but waned in April, and there were only three cases in May (Figure). There were three deaths, aged 59, 81 and 91 years. The cumulative incidence of 1,760 per 100,000 in Kupferzell by the end of April was, at the time of the study, one of the highest in Germany. The Robert Koch Institute (RKI) set out to analyse the SARS-CoV-2 seroprevalence in a random sample of this community from 20 May to 9 June.

**Figure:**
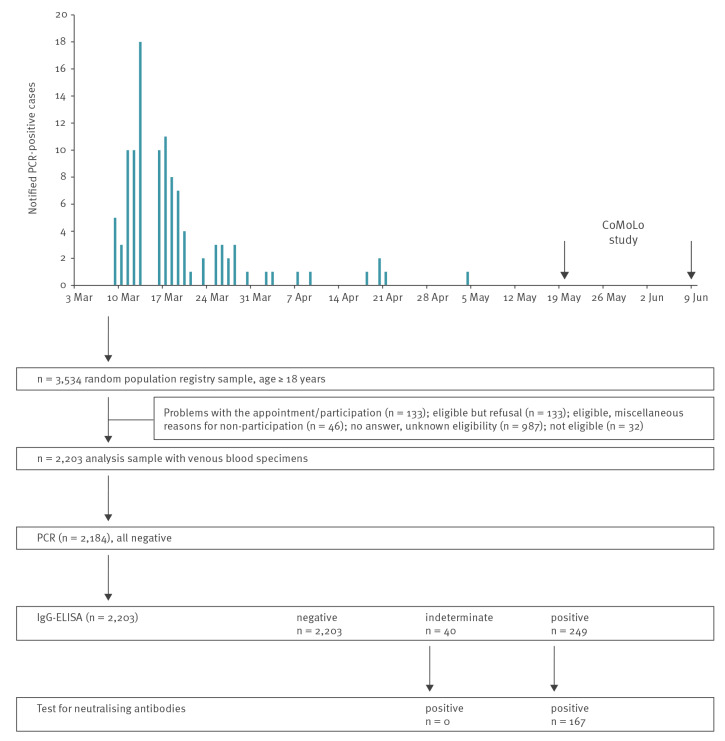
Notified COVID-19 cases in adults 18 years and older and flow-chart of study design, Kupferzell, Germany, March–June 2020 (n = 5,128)

COVID-19: coronavirus disease;

## CoMoLo study

The seroepidemiological study in Kupferzell, Germany is part of the population-based corona-monitoring local (CoMoLo) study that investigates the prevalence of SARS-CoV-2 IgG antibodies and of current infections in four communities with a high case incidence. Details are provided in the study protocol [1].

A random sample of 3,534 Kupferzell residents aged 18 years and older from the mandatory population registry (68.9% of the 5,128 adult residents) was invited to take part in the study, and 2,203; (48.5% women; 18–94 years; Table 1) had venous blood sampling (Figure). These participants were 63% of those eligible. Some 2,184 had SARS-CoV-2 RT-PCR testing of throat swabs targeting the E gene and the orf1ab region of SARS-CoV-2 [2]. The Robert Koch Institute performed SARS-CoV-2-S1 IgG-ELISA (Euroimmun, Lübeck, Germany) and applied the thresholds provided in the manual [1]. All samples that tested SARS-CoV-2-S1-IgG-positive (ratio ≥ 1.1) or indeterminate (ratio ≥ 0.8 to < 1.1) were additionally tested for neutralising antibodies with plaque reduction neutralisation tests (prNT) [3] at the German consultant laboratory for human coronaviruses at Charité – Universitätsmedizin Berlin.

**Table 1:** Characteristics of study population, COVID-19 cases 18 years and older, Kupferzell, Germany, 20 May–9 June 2020 (n = 2,203)

	n	Weighted %	95% CI
Sex			
Female	1,143	48.5	46.6–50.3
Male	1,060	51.5	49.7–53.4
Age female			
18–34 years	379	25.1	22.8–27.7
35–49 years	279	23.6	21.2–26.3
50–64 years	282	28.5	25.8–31.4
≥ 65	203	22.7	20.1–25.5
Age male			
18–34 years	334	26.6	23.8–29.6
35–49 years	254	26.2	23.3–29.3
50–64 years	290	28.2	25.6–31.1
≥ 65	182	18.9	16.5–21.6
Secondary school education			
Lower	670	42.8	40.3–45.3
Middle	731	28.2	26.4–30.1
Higher	744	29.0	26.9–31.1
Household size			
1 person	227	11.4	10.0–13.0
2 persons	729	34.3	31.7–36.9
3–4 persons	877	39.0	36.3–41.8
> 4 persons	324	15.3	13.2–17.8
Exposures			
Working with patients	204	10.0	8.6–11.5
Working with customers	432	21.3	19.3–23.3
Travelled abroad since 1 January	361	15.0	13.4–16.9
Participated in event with ≥ 50 persons	636	26.4	24.3–28.6
Quarantine or isolation			
Voluntary	256	11.8	10.3–13.4
Mandated	317	14.3	12.6–16.2
Self-reported health			
Very good	739	31.6	29.5–33.7
Good	1,156	55.2	52.9–57.4
Moderate/bad/very bad	247	13.2	11.7–14.9
Medical conditions			
Self-reported COVID-19	50	2.4	1.8–3.2
Chronic conditions^a^	638	35.3	33.0–37.7
Symptoms since 1 February			
Fever ≥ 38 °C	209	9.6	8.3–11.1
Dyspnoea, shortness of breath	145	6.6	5.6–7.9
Pneumonia	11	0.5	0.3–1.0
Congested/running nose	627	28.6	26.5–30.8
Cough	549	25.0	23.0–27.1
Pain when breathing	76	3.5	2.7–4.4
Sore throat	558	24.0	22.1–26.0
Loss of smell or taste	131	6.0	5.0–7.2
No symptoms	1,042	51.2	48.8–53.7
Mild symptoms only	922	41.8	39.4–44.2
Moderate or severe symptoms (pneumonia, dyspnoea)	153	7.0	5.9–8.3

CI: confidence interval; COVID-19: coronavirus disease.

^a^ Lung or heart disease, diabetes, stroke, hypertension, immunodeficiency.

Complementary categories are not always shown, e.g. ‘not working with patients’ and missing values are not shown, therefore n do not always add up to the total.

Underascertainment of SARS-CoV-2 infections was calculated as the ratio of two population proportions: the proportion of SARS-CoV-2 infections calculated from our study and the cumulative incidence of non-fatal PCR-positive cases in the adult population of Kupferzell calculated from notified cases aged 18 years and older. Proportions of IgG-positives were adjusted for sensitivity (88.3%) and specificity (99.2%) of the Euroimmun S1-SARS-CoV-2 IgG test [4], according to validity studies conducted by the Paul Ehrlich Institute. These validity studies had tested 513 pre-pandemic specimens and 222 convalescent coronavirus disease (COVID-19) patients, the vast majority (96%) at least 21 days after symptom onset (personal communication, H. Scheiblauer, 30 Sep 2020).

Statistical analyses were conducted using SAS 9.4 survey procedures. Results were weighted to the population of Kupferzell with regard to age group, sex and school education (district level). Clustering within households was taken into account.

## Ethical statement

This study was approved by the ethics committee of the Berlin Chamber of Physicians (Berliner Ärztekammer, reference number Eth-11/20), and the data commissioner of the Robert Koch Institute. All participants gave informed consent.

## Seroprevalence

All SARS-CoV-2 swabs taken during the study were negative in RT-PCR. The population-weighted prevalence of indeterminate IgG results was 1.9%; positive IgG results occurred with a prevalence of 11.3% or, when corrected for test performance, 12.0% (95% confidence interval (CI): 10.4–14.0) (Table 2). The lowest IgG seroprevalence in women was among the 18–34 year-olds, in men among the 35–49 year-olds. Factors associated with seropositivity were loss of smell or taste, fever ≥ 38 °C, a history of travelling or attending a large event and very good self-reported health. The association of seropositivity with ‘quarantine or isolation’ is not surprising since these participants were likely to be either diagnosed COVID-19 cases or close contacts. None of the participants with indeterminate IgG had a positive prNT, i.e. neutralising antibodies. The population-weighted seroprevalence of anti-SARS-CoV-2 IgG with positive prNT was 7.7% (95% CI: 6.5–9.1).

**Table 2:** Prevalence of SARS-CoV-2 IgG and neutralising antibodies in adults and association with sociodemographic, exposure and clinical characteristics, Kupferzell, Germany, 20 May–9 June 2020 (n = 2,203)

	Prevalence of positive results in both IgG-ELISA (ratio ≥ 1.1) and prNT			IgG-ELISA-positive (ratio ≥ 1.1)		Seroprevalence: prevalence of IgG ratio ≥ 1.1 corrected for sensitivity 88.3% and specificity 99.2%		OR for being IgG-seropositive adjusted for age group and sex		Distribution among seropositives^a^ (IgG-ELISA; n = 249)	
	n	Weighted %	95% CI	n	Prevalence, weighted %	Weighted %	95% CI	OR	95% CI	Weighted %	95% CI
**Total**	**167**	**7.7**	**6.5–9.1**	**249**	**11.3**	**12.0**	**10.4–14.0**	Nd		Nd	
Female	96	8.7	7.1–10.7	136	12.2	13.0	10.8–15.6	1	Reference	52.1	46.1–58.1
Male	71	6.7	5.2–8.5	113	10.5	11.1	9.0–13.6	0.86	0.67–1.12	47.9	41.9–53.9
Age female											
18–34 years	23	5.4	3.5–8.1	31	7.5	7.7	5.1–11.3	1	Reference	15.5	10.8–21.7
35–49 years	20	7.2	4.6–11.1	39	14.3	15.4	11.0–21.0	2.04	1.21–3.44	27.6	20.6–36.0
50–64 years	30	10.3	7.3–14.4	39	13.4	14.4	10.4–19.5	1.90	1.16–3.12	31.3	23.8–39.9
≥ 65	23	12.0	8.1–17.5	27	13.7	14.8	10.0–21.2	1.95	1.12–3.42	25.5	18.2–34.5
Age male											
18–34 years	25	7.2	4.7–10.8	40	11.3	12.0	8.2–17.1	1	Reference	28.6	20.7–38.1
35–49 years	6	2.4	1.0–5.3	11	4.5	4.3	1.9–8.4	0.37	0.18–0.78	11.2	6.2–19.6
50–64 years	23	8.1	5.4–12.0	37	12.9	13.9	9.9–19.0	1.16	0.70–1.93	34.7	26.2–44.3
≥ 65	17	9.8	6.1–15.3	25	14.2	15.3	10.2–22.3	1.30	0.73–2.32	25.5	17.6–35.4
Secondary school education											
Lower	61	9.0	6.7–11.8	82	11.6	12.3	9.7–15.5	0.92	0.62–1.35	43.3	36.5–50.3
Middle	58	8.4	6.2–11.2	86	12.2	13.0	10.3–16.3	1.10	0.78–1.55	30.0	24.5–36.1
Higher	46	6.0	4.2–8.6	78	10.6	11.2	8.6–14.4	1	Reference	26.8	21.5–32.8
Household size											
1 person	14	6.8	4.0–11.3	21	9.6	10.1	6.2–15.7	0.79	0.46–1.37	9.6	6.2–14.5
2 persons	57	8.0	6.0–10.6	82	11.5	12.2	9.5–15.5	1	Reference	34.5	27.9–41.8
3–4 persons	71	8.3	6.4–10.7	106	12.1	12.9	10.2–16.2	1.29	0.90–1.84	41.5	34.4–48.9
> 4 persons	23	6.2	3.7–10.2	37	10.7	11.4	7.1–17.4	1.13	0.67–1.90	14.4	9.5–21.2
Exposures											
Working with patients	23	12.0	8.0–17.6	30	14.9	16.1	10.9–23.0	1.41	0.90–2.22	13.3	9.3–18.8
Working with customers	31	7.7	5.2–11.1	48	11.9	12.7	9.2–17.2	1.16	0.79–1.71	22.7	17.1–29.5
Travelled abroad since 1 January	31	8.9	5.8–13.5	58	16.7	18.1	13.3–24.2	1.93	1.31–2.83	21.9	16.4–28.7
Event with ≥ 50 persons	68	11.9	9.3–15.2	102	17.2	18.8	15.0–23.1	2.24	1.63–3.07	39.8	33.2–46.7
Quarantine or isolation											
Voluntary	28	11.8	8.1–16.9	40	17.3	18.9	13.7–25.5	3.34	2.17–5.15	18.4	13.5–24.5
Mandated	80	25.4	20.5–31.1	104	33.1	36.9	30.4–44.1	8.68	6.00–12.55	42.5	35.4–50.0
Self-reported health											
Very good	57	7.7	5.9–10.0	95	13.1	14.0	11.2–17.5	1.41	1.04–1.90	36.0	30.0–42.4
Good	92	8.1	6.5–10.1	127	11.1	11.8	9.6–14.3	1	Reference	53.2	46.8–59.6
Moderate/bad/very bad	15	6.3	3.8–10.2	23	9.4	9.8	6.2–14.9	0.69	0.42–1.14	10.8	7.2–15.8
Medical conditions											
Self-reported COVID-19	34	71.6	57.8–82.3	43	89.0	100.8	88.0–107.5	81.20	34.78–189.55	19.2	14.2–25.4
Chronic conditions^b^	53	8.9	6.7–11.7	71	11.6	12.3	9.5–15.8	0.78	0.55–1.10	35.4	28.9–42.4
Symptoms since 1 February											
Fever ≥ 38 °C	66	32.7	26.0–40.3	77	38.4	42.9	34.8–51.7	6.82	4.78–9.72	31.4	25.4–38.2
Dyspnoea, shortness of breath	28	19.4	13.4–27.3	36	25.8	28.5	20.6–38.2	2.80	1.81–4.33	14.6	10.5–20.1
Pneumonia	Nd			4	Nd	Nd		Nd		Nd	
Congested/running nose	74	11.6	9.0–14.8	102	16.0	17.3	13.9–21.4	1.88	1.39–2.56	39.0	32.7–45.7
Cough	76	14.1	11.1–17.7	101	18.6	20.4	16.5–25.0	2.34	1.73–3.17	39.9	33.5–46.7
Pain when breathing	13	17.0	10.0–27.5	17	22.9	25.3	15.4–38.6	2.39	1.31–4.36	6.8	4.2–10.9
Sore throat	55	10.1	7.6–13.1	68	12.5	13.3	10.2–17.2	1.20	0.86–1.68	25.7	20.3–31.9
Loss of smell or taste	69	54.9	45.8–63.7	92	71.5	80.8	70.8–89.3	30.49	19.68–47.25	36.5	30.2–43.3
No symptoms	24	2.6	1.7–4.0	55	5.6	5.5	3.9–7.6	1	Reference	24.5	18.9–31.1
Mild symptoms only	113	12.6	10.3–15.4	152	16.6	18.1	15.0–21.6	3.77	2.62–5.42	59.4	52.4–66.1
Moderate or severe symptoms (pneumonia, dyspnoea)	29	19.2	13.3–26.8	39	26.8	29.7	21.8–39.2	6.30	3.85–10.30	16.1	11.7–21.7

CI: confidence interval; COVID-19: coronavirus disease; ELISA: enzyme-linked immunosorbent assay; Nd: not done; OR: odds ratio; prNT: plaque reduction neutralisation tests; SARS-CoV-2: severe acute respiratory syndrome coronavirus 2.

^a^ Not corrected for sensitivity and specificity.

^b^ Lung or heart disease, diabetes, stroke, hypertension, immunodeficiency.

Complementary categories are not always shown, e.g. ‘not working with patients’ and missing values not shown, therefore n do not always add up to the total.

## Cumulative incidence of SARS-CoV-2 infections

For the cumulative incidence of SARS-CoV-2 infections, we considered current infections (in this study none because all study PCR tests were negative) and past infections. The vast majority of past infections can be identified by IgG antibodies, but not all [5]: in the subgroup of 50 participants with self-reported COVID-19 diagnosis done before the study period, only 89% (weighted percentage; 95% CI: 77.3–95.0) were IgG-positive (Table 3). The seropositivity rate in the 26 participants with self-reported COVID-19 diagnosis with mild symptoms was 87% (weighted percentage; 95% CI: 70.7–95.1) and in those with moderate-to-severe symptoms (n = 16) it was 94% (weighted percentage; 95% CI: 66.5–99.3). However, this was well taken into account by the mathematical correction for sensitivity and specificity since the corrected proportion of seropositives among these 50 participants was ca 100%. 24.5% of seropositive participants reported that they had not had any of the eight investigated symptoms since 1 February (16.8% of those with neutralising antibodies).

**Table 3:** Participants with self-reported COVID-19 diagnosis, Kupferzell, Germany, 20 May–9 June 2020 (n = 50)

	Total			18–49 years			≥ 50 years		
Total (n unweighted)	50			25			25		
Mean age in years (range)	52 (19–81)			37 (19–49)			63 (50–81)		
	n	Column % ^a^	95% CI	n	Column % ^a^	95% CI	n	Column % ^a^	95% CI
IgG-positive	43	89.0	77.3–95.0	19	79.1	58.6–91.0	24	95.9	74.6–99.5
IgG-positive, corrected for sensitivity 88.3% and specificity 99.2%	43	100.8	87.4–107.7	19	89.5	66.0–103.1	24	108.7	84.3–112.8
IgG-positive and prNT-positive	34	71.6	57.3–82.6	13	54.6	34.3–73.5	21	83.5	62.5–93.9
Chronic conditions^b^	19	45.8	31.5–60.9	6	29.5	13.2–53.6	13	57.7	36.5–76.5
No symptoms	2	Nd		2	Nd		0	Nd	
Mild symptoms only	31	61.4	45.9–74.9	18	70.6	47.7–86.3	13	55.0	34.9–73.6
Moderate-to-severe symptoms (pneumonia, dyspnoea/shortness of breath)	17	34.4	21.6–49.9	5	19.2	7.7–40.6	12	45.0	26.4–65.1

CI: confidence interval; COVID-19: coronavirus disease; Nd: not done; OR: odds ratio; prNT: plaque reduction neutralisation tests; SARS-CoV-2: severe acute respiratory syndrome coronavirus 2.

^a^ Weighted %.

^b^ Lung or heart disease, diabetes, stroke, hypertension, immunodeficiency.

There were no indeterminate IgG results. Of note, while n are unweighted, proportions are weighted and can therefore not be calculated from the numbers in this table.

The underascertainment ratio comparing IgG seropositivity corrected for test performance, with the officially reported cumulative incidence was 6.1 (95% CI: 5.2–7.0). If calculated based on seropositivity of both IgG and prNT, the underascertainment ratio would be 3.9 (95% CI: 3.2–4.6).

## Discussion

Seroepidemiological studies are key to understanding the distribution of infections in the population, despite uncertainties deriving from test performance and from the proportion of infected persons who never develop or have declining levels of antibodies [5-10].

Our results of 12% IgG-seropositive participants corrected for test performance and a proportion of 25% asymptomatic infections are in line with the results from the German high-prevalence towns Gangelt [10] and Neustadt am Rennsteig [11]. Seroepidemiological studies conducted in Germany [12] are systematically tracked by the German national public health institute (Robert Koch Insitute; www.rki.de/covid-19-serostudies-germany). The cumulative incidence of infections of 15.5% in Gangelt [13] was based on RT-PCR-positive cases and on positive or indeterminate S1-ELISA-Euroimmun IgG tests, corrected for the manufacturer-provided sensitivity of 90.9% and specificity of 99.1%. From Neustadt am Rennsteig [11], a seroprevalence of 8.4% was reported, based on two of six different IgG immunoassays. Testing of a pre-existing population-based cohort in the low-prevalence area of Bonn yielded a seroprevalence of ca 1%, based on positive S1-ELISA-Euroimmun IgG tests and 0.36% with both S1-ELISA-IgG and neutralising antibodies [14]. Compared with other European areas with high COVID-19 prevalence such as Ischgl in Austria [15] or the Lodi Red Zone in Lombardy, Italy, [16] the seroprevalence in Kupferzell was still low.

The increased odds of infection after travelling abroad and after participating in larger events are in line with the outbreak history in Kupferzell. From our study and the three other German studies with available data, the underascertainment ratio has been smaller than 6 [11,13,14] and not 10 or higher as in a number of international locations [17]. The association of seropositivity with very good self-reported health, although not statistically significant, may be indicative of lower risk awareness and less protective behaviour. As the CoMoLo study continues in three other locations, more detailed analyses might be possible with a larger sample.

According to a recent report of IgG levels stable for up to 4 months on the one hand [18], and reports on waning of neutralising antibodies on the other hand [10,19,20], we base our estimate of the cumulative incidence of infections on IgG antibodies. However, in our subsample of 50 participants with self-reported PCR-based COVID-19 diagnoses, 11% were not IgG-positive which is in line with large population-based studies from Spain and New York State [21,22]. The cumulative incidence of infection in this subgroup, which was based on IgG corrected for sensitivity and specificity, took these seronegative infected persons almost perfectly into account. However, with increasing time lag between pandemic wave and serosurveys, some additional adjustment for seroreversion may be necessary when estimating the cumulative incidence. Of note, validation studies for serological assays should have sufficient sample sizes in the healthy group, where specificity is calculated, and in the infected group, where sensitivity is calculated. In addition, they should aim for representativeness of the target population as well as clinical outcome (mild and severe COVID-19) and address cross-reactivity concerns by including subgroups of patients with other respiratory virus infections including seasonal cororavirus [23].

In Neustadt am Rennsteig, only 20 of 38 (53%; 95% CI: 37–69) previously PCR-positive persons were seropositive, which may be due to a different testing strategy (whole community screening) that tested more asymptomatic cases and to the definition of seropositivity (at least two of six different antibody tests needed to be positive). Therefore, seronegative infected persons may not have been taken into account sufficiently and the underlying cumulative incidence of infections may have been as high as 8.4 per 0.52, i.e. 16%. We therefore propose that estimates of the cumulative incidence of infections should be based not only on antibody testing but also on current and past PCR test results. Within each study, the subsample of previously PCR-positive participants, i.e. participants for whom serological and virological results are available, provides valuable information for estimating the cumulative incidence of infections. It can be used to evaluate whether correction for diagnostic sensitivity, e.g. mathematical correction or combination of different immunoassays, is appropriate for the specific study.

## Conclusion

This study confirmed that even in areas with high COVID-19 prevalence, only a small proportion of the population has been infected. Therefore, ongoing protective measures are justified. Moreover, this is the second German study on a community outbreak that shows that these measures are highly effective, leading at least temporarily to full containment [11].
